# NanoString technology distinguishes anti‐TIF‐1γ^+^ from anti‐Mi‐2^+^ dermatomyositis patients

**DOI:** 10.1111/bpa.12957

**Published:** 2021-05-27

**Authors:** Corinna Preusse, Pascale Eede, Lucie Heinzeling, Kiara Freitag, Randi Koll, Waltraud Froehlich, Udo Schneider, Yves Allenbach, Olivier Benveniste, Anne Schänzer, Hans‐Hilmar Goebel, Werner Stenzel, Josefine Radke

**Affiliations:** ^1^ Department of Neuropathology Charité‐Universitätsmedizin Berlin, corporate member of Freie Universität Berlin, Humboldt‐Universität zu Berlin and Berlin Institute of Health Berlin Germany; ^2^ Department of Neurology with Institute for Translational Neurology Münster University Hospital (UKM) Münster Germany; ^3^ Department of Dermatology University Hospital of Erlangen Erlangen Germany; ^4^ Department of Dermatology LMU Munich Germany; ^5^ German Center for Neurodegenerative Diseases (DZNE) within the Helmholtz Association Berlin Germany; ^6^ German Cancer Consortium (DKTK) Berlin Germany; ^7^ Department of Rheumatology and Clinical Immunology Charité‐Universitätsmedizin Berlin, corporate member of Freie Universität Berlin, Humboldt‐Universität zu Berlin and Berlin Institute of Health Berlin Germany; ^8^ Department of Internal Medicine and Clinical Immunology Sorbonne Université, Pitié‐Salpêtrière University Hospital Paris France; ^9^ Department of Neuropathology Justus Liebig Universität Giessen Giessen Germany; ^10^ Berlin Institute of Health (BIH) Berlin Germany

**Keywords:** dermatomyositis, Mi‐2, myositis‐specific antibody, NanoString, skeletal muscle, TIF‐1γ

## Abstract

Dermatomyositis (DM) is a systemic idiopathic inflammatory disease affecting skeletal muscle and skin, clinically characterized by symmetrical proximal muscle weakness and typical skin lesions. Recently, myositis‐specific autoantibodies (MSA) became of utmost importance because they strongly correlate with distinct clinical manifestations and prognosis. Antibodies against transcription intermediary factor 1γ (TIF‐1γ) are frequently associated with increased risk of malignancy, a specific cutaneous phenotype and limited response to therapy in adult DM patients. Anti‐Mi‐2 autoantibodies, in contrast, are typically associated with classic DM rashes, prominent skeletal muscle weakness, better therapeutic response and prognosis, and less frequently with cancer. Nevertheless, the sensitivity of autoantibody testing is only moderate, and alternative reliable methods for DM patient stratification and prediction of cancer risk are needed. To further investigate these clinically distinct DM subgroups, we herein analyzed 30 DM patients (n = 15 Mi‐2^+^ and n = 15 TIF‐1 γ^+^) and n = 8 non‐disease controls (NDC). We demonstrate that the NanoString technology can be used as a very sensitive method to clearly differentiate these two clinically distinct DM subgroups. Using the nCounter PanCancer Immune Profiling Panel™, we identified a set of significantly dysregulated genes in anti‐TIF‐1γ^+^ patient muscle biopsies including *VEGFA*, *DDX58*, *IFNB1*, *CCL5*, *IL12RB2*, and *CD84*. Investigation of type I IFN‐regulated transcripts revealed a striking type I interferon signature in anti‐Mi‐2^+^ patient biopsies. Our results help to stratify both subgroups and predict, which DM patients require an intensified diagnostic procedure and might have a poorer outcome. Potentially, this could also have implications for the therapeutic approach.

## Graphical abstract

Dermatomyositis (DM) is a systemic idiopathic inflammatory disease affecting skeletal muscle and skin, clinically characterized by symmetrical proximal muscle weakness and typical skin lesions. Genetic profiling of anti‐TIF‐1γ^+^ and ‐Mi‐2^+^ dermatomyositis patients’ skeletal muscle biopsies revealed subgroup‐specific signatures, which helps to stratify both subgroups and predict, which DM patients require an intensified diagnostic procedure and might have a poorer outcome.

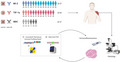

## 1 INTRODUCTION

Dermatomyositis (DM) is a rare idiopathic inflammatory disease of the skeletal muscle and skin with heterogeneous clinical and morphological presentation. Histomorphologically, inflammatory infiltrates of various immune cells, perifascicular atrophy, specific injury to capillaries and perifascicular myofibers, and MHC‐I upregulation are common in DM (1, 2). Clinically, the presence of muscle weakness is mostly associated with skin symptoms like Gottron's papules, Gottron's sign, and/or the heliotrope eruption (2, 3). New biomarkers, such as DM‐specific autoantibodies (anti‐Mi‐2, anti‐MDA5, anti‐TIF‐1γ, anti‐NXP2, and anti‐SAE) correlate with distinct clinical phenotypes with respect to organ involvement and malignancy in cancer‐associated myositis (CAM, (4)). The association of anti‐TIF‐1γ antibody and increased risk of cancer in adult DM has recently been highlighted (4, 5) and characteristic cutaneous findings, such as palmar hyperkeratotic papules, psoriasis‐like lesions, and hypopigmented and telangiectatic "red‐on‐white" patches have been described (6). TIF‐1γ (TRIM33) plays an important role in carcinogenesis and cell differentiation (7, 8). Cancer development, including various entities e.g. breast, colorectal and ovarian cancer, as well as Hodgkin's lymphoma (9, 10, 11, 12) may be recognized years before or after diagnosis of DM (13). Anti‐TIF‐1γ‐associated DM typically presents with proximal limb weakness accompanied by severe skin changes, moderately elevated CK levels, and absence of interstitial lung disease (ILD) (7). In muscle biopsies, complement (C5b‐9) deposition on intramuscular capillaries has been shown to be associated with malignancy (1, 5). Proximal muscle weakness may also be seen in anti‐Mi‐2^+^ DM patients in which classical Gottron's papules or heliotrope rash are more common and CK levels are usually high, whereas other organ involvement is generally absent. Histomorphologically, anti‐Mi‐2‐associated DM typically demonstrates characteristic perifascicular atrophy, necrotic myofibers, prominent inflammatory infiltrates, and MHC‐I expression on perifascicular fibers. Anti‐Mi‐2^+^ DM patients respond well to standard treatment, including corticosteroids and rituximab and show a better overall prognosis (14, 15, 16).

An autoantibody‐based distinction of these two clinically distinct subsets of adult DM is important for further patient stratification and follow‐up. Nevertheless, the detection of autoantibodies in DM is not well‐standardized (2, 4, 17). There are no international reference samples available so far, whereas different therapeutic regimen such as administration of B‐cell directed therapy (rituximab), plasmapheresis, or administration of intravenous immunoglobulins may influence the concentration of autoantibodies and subsequently decreased disease activity (2). Therefore, additional robust and reliable methods are needed. Here, we utilize the NanoString‐based nCounter PanCancer Immune Profiling Panel™ (18), quantifying the expression levels of 770 genes related to immune‐oncological signaling pathways and cell types to characterize muscle biopsies from DM patients harboring anti‐TIF‐1γ and anti‐Mi‐2 autoantibodies, to see if any DM‐specific subgroups may be differentiated using an alternative molecular technique. Special interest was given to these two DM subgroups as prognosis, association with cancer and treatment response differ substantially. In addition, we wanted to i) investigate if cancer association in DM may be detected by this technique and ii) further analyze subgroup‐specific expression profiles to better understand the pathogenesis and potential risk factors for cancer development.

## 2 METHODS

### 2.1 Patient cohort

**FIGURE 1:**
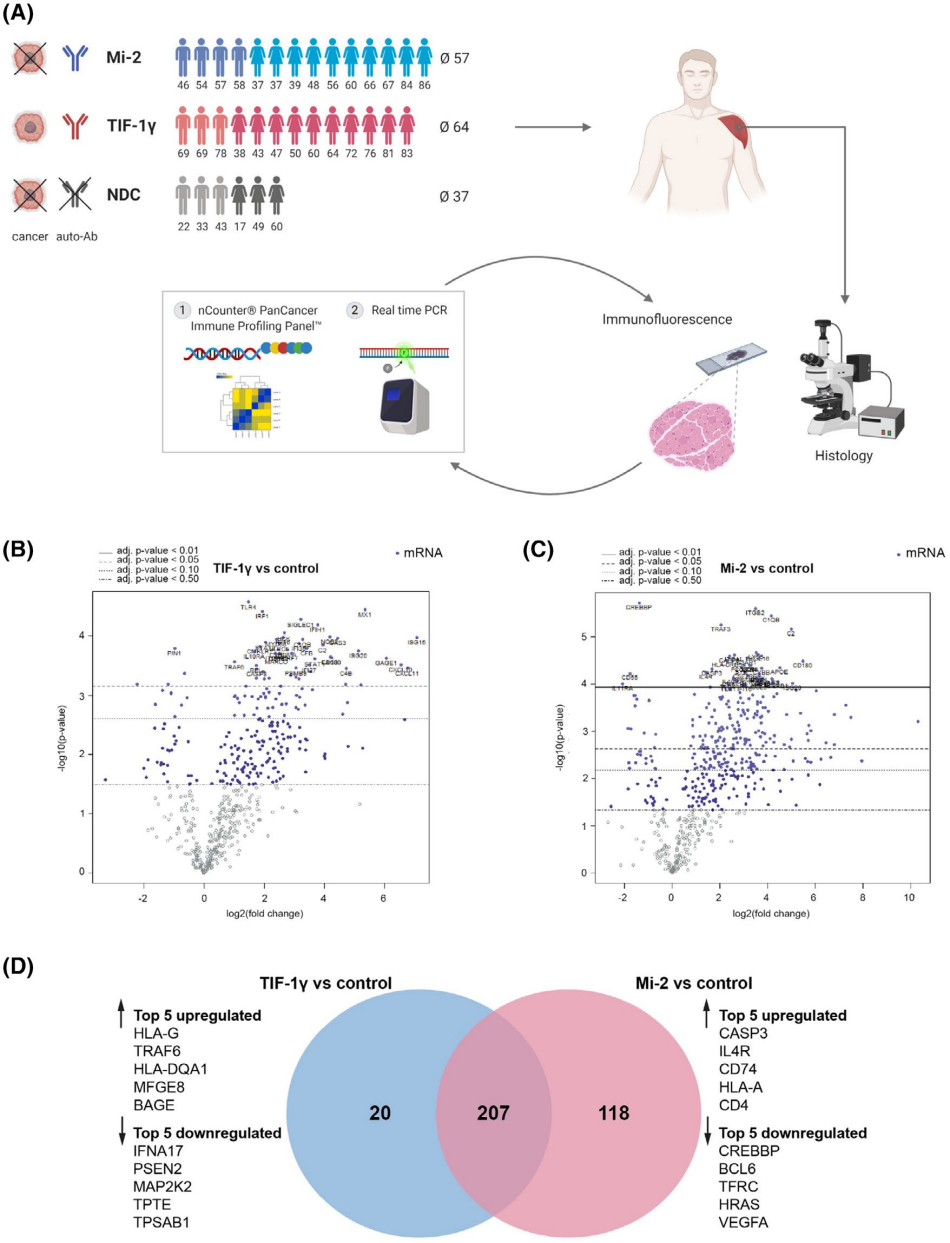
Genetic profiling of anti‐TIF‐1γ^+^ and ‐Mi‐2^+^ dermatomyositis patients’ skeletal muscle biopsies revealed subgroup‐specific signatures. (A) Experimental design and analysis workflow of the project. (B) Differential gene expression analysis from anti‐TIF‐1γ^+^ patients (n = 6) and non‐diseased controls (n = 2). (C) Differential gene expression analysis from anti‐Mi‐2^+^ patients (n = 6) and non‐diseased controls (n = 2). (D) Venn diagram comparing the differentially expressed genes (in skeletal muscle tissues) of anti‐TIF‐1γ^+^ patients vs. non‐diseased controls and anti‐Mi‐2^+^ patients vs. non‐diseased controls, identifying 207 commonly dysregulated genes, 20 TIF‐1γ subgroup‐specific genes and 118 Mi‐2 subgroup‐specific genes. The top 5 up‐ and downregulated genes specific for each subgroup are highlighted

The study cohort and sample size as well as the experimental design, analysis workflow, diagnosis, and autoantibody status are displayed in Figure 1A and Table S1. The available clinical and demographic information of all (n = 38) patients enrolled in this study are listed in Table S1. Skeletal muscle biopsies were analyzed from patients diagnosed (according to EULAR classification criteria) with DM and positive autoantibodies, against Mi‐2 (n = 15, using n = 6 for NanoString, n = 11 for qPCR, and n = 7 for histology) or TIF‐1γ (n = 15, using n = 6 for NanoString, n = 9 for qPCR, and n = 7 for histology). Cancer‐associated myositis (CAM) was present in n = 9 (60%) anti‐TIF‐1γ^+^ and n = 3 (20%) anti‐Mi‐2^+^ patients. CAM was defined as neoplasms occurring within 2 years before or no more than 3 years after myositis onset as previously described (2, 5). Patients that suffered from cancer more than 2 years before or more than 3 years after DM diagnosis were defined as CAM‐. Patients labeled “no cancer” never suffered from cancer at all.

Additionally, we investigated skeletal muscle biopsies (n = 8, using n = 2 for NanoString, n = 3 for qPCR and n = 6 for histology) from patients with nonspecific complaints in the context of “fatigue‐like” symptoms, without clinical muscle weakness, with the absence of any morphologic abnormalities on skeletal muscle biopsies, of elevated creatine kinase (CK) levels, or laboratory evidence of any systemic inflammation serving as non‐diseased controls (NDC).

### 2.2 Standard protocol approvals and patient consents

Informed consent was obtained from all patients at each institution involved. Procedures were approved by the official ethical standards committee (EA2/163/17) at the Charité‐Universitätsmedizin Berlin.

### 2.3 Histologic, immunohistochemical, and immunofluorescence procedures

Histological stains were performed on 7‐μm cryostat muscle sections according to standard procedures. Immunohistochemical and double immunofluorescence stains with antibodies against laminin‐α5 and VEGF, nMyHc and VEGF, DDX58/RIG‐1 and CD68, MSTR1R/RON and CD31 as well as pRON and nMyHc, PDGFRβ, MHC class II, CD8, CD206, and CD4 were performed as previously described (19, 20). Primary antibodies are listed in Table S2.

### 2.4 RNA isolation and cDNA transcription

Total RNA was isolated from muscle specimens using TRIzol™ Reagent (Thermo Fisher Scientific, Germany) as previously described (20). Complementary DNA (cDNA) was synthesized using the High‐Capacity cDNA Archive Kit (Applied Biosystems, Foster City, CA, USA) according to the manufacturer's protocol.

### 2.5 Quantitative real‐time PCR (qPCR)

We performed quantitative real‐time PCR (qPCR) measuring the gene expression profile of *VEGF*, *DDX58*, and *MSTR1* using the following TaqMan probes (ThermoFisher Scientific): Hs00900055_m1 (*VEGFA*), Hs00204833_m1 (*RIG1*/*DDX58*), Hs00899920_m1 (*MST1R*), and Hs99999905_m1 (*GAPDH*). Glyceraldehyde 3‐phosphate dehydrogenase (GAPDH) was included as an internal control to normalize the expression of the target genes. qPCR analysis was performed using an Applied Biosystems™ QuantStudio™ 6 Flex Real‐Time PCR System (ThermoFischer, Waltham, MA; USA) with the following thermal profile: 20 s at 95°C, followed by 40 cycles of 1 s at 95°C and 20 s at 60°C.

### 2.6 NanoString analysis

We analyzed the expression of 770 genes (including 40 reference genes) related to the immune response in cancer using the nCounter PanCancer Immune Profiling Panel™ (human) (Nanostring, XT‐CSO‐HIP1‐12). 200–500 ng of total RNA was used as input and sample hybridization was performed according to the manufacturer's instructions. Sample detection and analysis were completed on a nCounter® Digital Analyzer. Raw data processing, quality control, and normalization were performed using the nSolver™ 4.0 analysis software. Quality control (QC) and normalization were performed with an imaging QC of >75% field of view registration, binding density QC within 0.1‐2.25 range, positive control linearity QC of R^2^ above 0.95, and positive control limit of detection set as 0.5 fM positive control above 2 standard deviations above the mean of the negative controls. Normalization to housekeeping genes, of which genes below 100 were excluded, and differential expression analysis were completed using the Advanced Analysis software plugin (version 2.0.115). For differential expression analysis, a log2 fold change of ≤−1 or ≥1 as well as a *p*‐value of ≤0.05 were applied as cutoffs. For the first analysis, gene expression measurements from anti‐Mi‐2^+^ patient muscle biopsies (n = 6) and anti‐TIF‐1γ^+^ patients’ muscle biopsies (n = 6) were normalized to healthy non‐diseased control samples (NDC, n = 2) before being compared to each other. For the second analysis, anti‐Mi‐2^+^ patients’ muscle biopsies (n = 6) were directly compared to anti‐TIF‐1γ^+^ patients’ biopsies (n = 6).

### 2.7 Evaluation of NanoString results

To further analyze the associated pathways of the differentially expressed genes, functional enrichment analysis was performed using Enrichr (21, 22) for Gene Ontology (GO) to identify the annotated sets of genes based on the biological processes in which they participate.

### 2.8 Statistical analysis

Non‐parametric Kruskal‐Wallis one‐way analysis of variance followed by multiple comparison was used for gene transcript analysis. Data are presented as mean ± SD. The level of significance was set at *p* < 0.05. GraphPad Prism 9.0.0 software (GraphPad Software, Inc., La Jolla, CA) was used for statistical analysis.

## 3 RESULTS

### 3.1 Genetic profiling of dermatomyositis patients revealed subgroup‐specific signatures

In order to identify disease‐specific gene signatures in DM subgroups, we performed NanoString gene expression analysis with RNA isolated from muscle biopsies obtained from patients with anti‐TIF‐1γ^+^ and anti‐Mi‐2^+^ antibodies as well as non‐diseased controls (NDCs). Compared to NDC biopsies from both anti‐TIF‐1γ^+^ and anti‐Mi‐2^+^ patients revealed strong dysregulation of immune response‐related genes. We detected 207 deregulated genes that were shared among both subgroups (Table S3). Gene Ontology (GO) term enrichment using Enrichr (21, 22) involved processes related to cytokine‐mediated signaling, T‐cell chemotaxis, type I interferon signaling, and inflammatory response. In fact, among these genes were many well‐known type 1 IFN‐inducible genes identified‐using INTERFEROME v2.01 (23)‐such as *ISG15*, *ISG20*, *MX1*, *STAT1*, *SIGLEC1*, *CXCR4*, *CCL19*, *CARD16*, and *IRF7* (Figure 1B,C, Table S3).

Additionally, we identified genes that were specifically deregulated in one of the two distinct subgroups in comparison to NDCs (Figure 1D, Table S3). Among the 20 specifically deregulated genes in anti‐TIF‐1γ^+^ patients’ skeletal muscles, the top five upregulated genes were *HLA*‐*G*, *TRAF6*, *HLA*‐*DQA1*, *MFGE8*, and *BAGE*, among the top 5 downregulated genes was *TPTE* (*PTEN2*), which shows homology to the tumor suppressor *PTEN*/*MMAC1* (Figure 1D). In anti‐Mi‐2^+^ skeletal muscles, 118 genes were specifically deregulated. The top five upregulated genes included *CASP3*, *IL4R*, *CD74*, *HLA*‐*A*, and *CD4* (Figure 1D), which were all classified as type 1 IFN‐inducible genes (INTERFEROME v2.01). Among the top 5 downregulated genes were *SH2D1B* (*EAT*‐*2*), *RORA*, *TFRC* (*CD71*), and *BCL6*, which are involved in differentiation and activation of regulatory T‐cells (24, 25, 26, 27) leading to suppression of inflammation (28).

### 3.2 NanoString analysis clearly distinguishes anti‐TIF‐1γ^+^ from anti‐Mi‐2^+^ dermatomyositis patients

**FIGURE 2:**
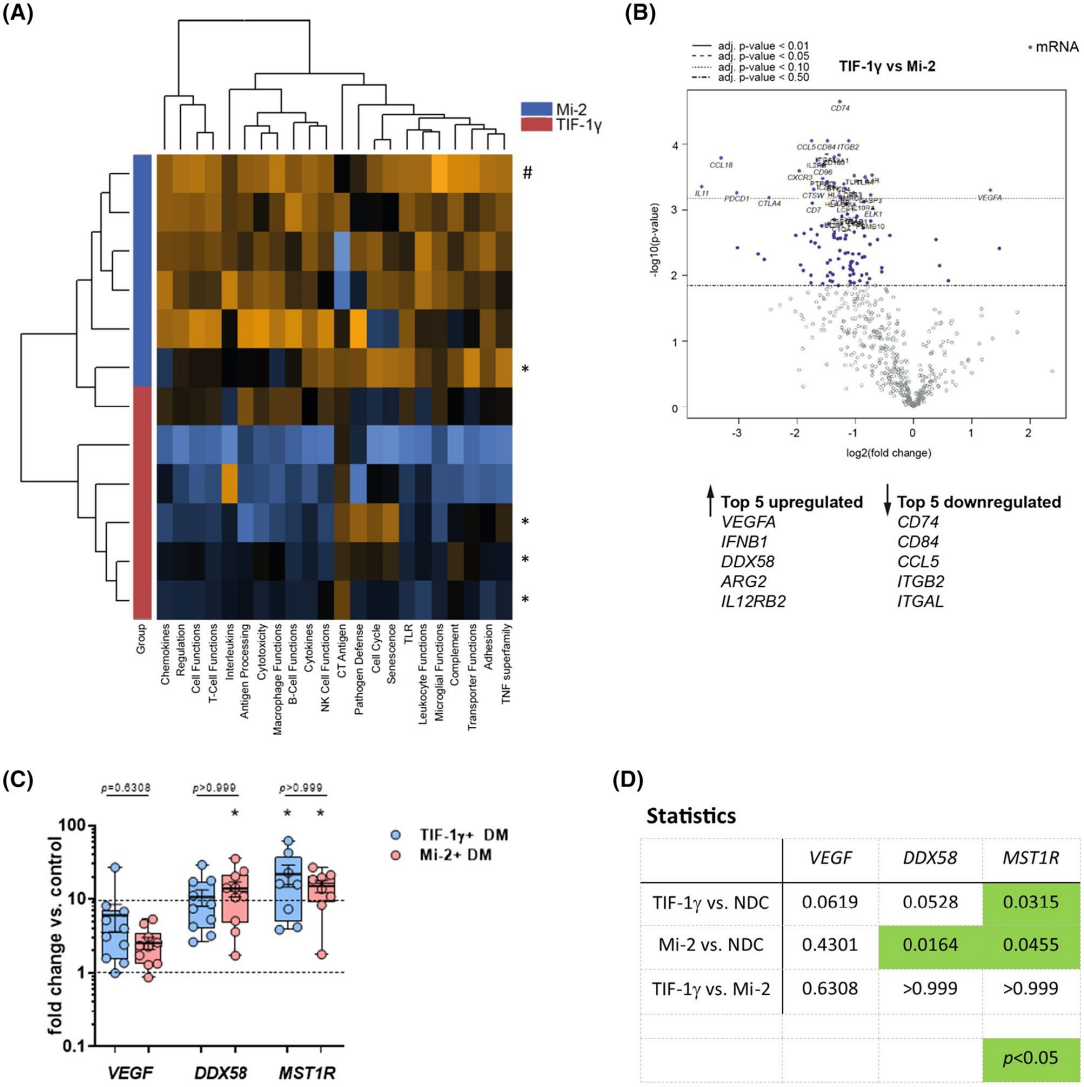
NanoString^®^ analysis clearly distinguishes anti‐TIF‐1γ^+^ from ‐Mi‐2^+^ dermatomyositis patients. (A) Unsupervised clustering of patient samples using the NanoString^®^ pathway score analysis tool. Asterisk (*) indicate CAM+ patients. Hashtag (#) indicates CAM‐ patient. (B) Differential gene expression analysis of anti‐TIF‐1γ^+^ patients vs. ‐Mi‐2^+^ patients. The top 5 up‐ and downregulated genes are highlighted. (C) Gene expression levels detected by qPCR of DM patients (each subgroup n = 10) displayed as fold‐change vs. NDC (n = 3), upregulation is significant for *DDX58*, *MST1R* in anti‐Mi‐2^+^ patients as well as for *MST1R* in anti‐TIF‐1γ^+^ patients, no significance between both subgroups was detected (C, D)

To further assess whether NanoString‐based gene profiling can readily differentiate between anti‐TIF‐1γ^+^‐ and anti‐Mi‐2^+^‐associated DM, we performed a pathway score analysis, which functionally annotated groups of genes followed by unsupervised clustering of samples. Here, we found a clear separation of both DM groups into two distinct clusters (Figure 2A). Interestingly, the one Mi‐2^+^/CAM+ case clustered closer with the TIF‐1γ^+^ cases than the other cancer‐free patients. Within the group of TIF‐1γ^+^ patient samples, CAM+ cases clustered together (Figure 2A, asterisks) indicating an additional, CAM‐associated expression profile. A comparison of the differentially expressed genes in anti‐TIF‐1γ^+^ versus anti‐Mi‐2^+^ patients’ biopsies revealed a distinct subgroup‐specific gene signature (Figure 2B, Table S2). The top 5 upregulated genes in anti‐TIF‐1γ^+^ patients’ muscle compared to anti‐Mi‐2^+^ patients’ skeletal muscles were: *VEGFA*, *IFNB1*, *DDX58*, *ARG2*, and *IL12RB2*. The top 5 downregulated genes in TIF‐1γ^+^ patients' muscle compared to Mi‐2^+^ patients' muscle were: *CD74*, *CD84*, *CCL5*, *ITGB2*, and *ITGAL* (Figure 2B). To further elucidate the molecular mechanisms of anti‐TIF‐1γ autoantibody and association to cancer in adult DM patients‐since an association with cancer was described in the literature, we focused on specifically dysregulated genes in anti‐TIF‐1γ^+^ patients compared to NDCs or anti‐Mi‐2^+^ individuals. We could identify *VEGFA* (29, 30), *BAGE* (31, 32), *DDX58* (RIG‐I, (33)), and *MST1R (RON*, (34)) as upregulated and *TPTE* (35) as downregulated. As shown in Figure 2C, the expression levels of *VEGF*, *DDX58/RIG‐1*, and *MST1R/RON* were increased in comparison to NDCs, in line with the NanoString results. The expression reached significance for *DDX58* and *MSTR1* in both subgroups.

For validation of our findings, with qPCR we analyzed expression levels in additional muscle samples of anti‐TIF‐1γ^+^ and anti‐Mi‐2^+^ patients. However, *VEGF*, *DDX58*, and *MST1R* expression showed no significant differences between the two subgroups (Figure 2D), which is in line with previous reports demonstrating that NanoString showed superior sensitivity compared to that of qPCR (36, 37). NanoString is, therefore, better suited to identify subtle differences in the gene expression levels.

### 3.3 Protein expression of VEGF and DDX58/RIG‐1 is enhanced in perifascicular areas

**FIGURE 3:**
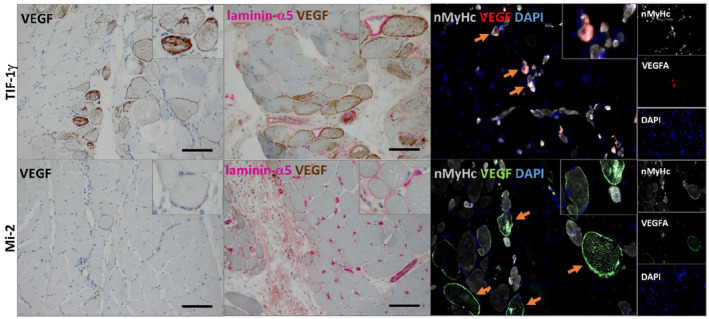
Histological and immunofluorescent staining of VEGF in DM patients’ skeletal muscle samples. Anti‐TIF‐1γ^+^ DM patients show clearly enhanced expression of sarcolemmal VEGF in the perifascicular areas while anti‐Mi‐2^+^ DM patients show only single positive fibres (left panel). Double immune histochemistry identified multiple VEGF^+^ myofibres and a severe depletion of laminin‐α5^+^ capillaries in anti‐TIF‐1γ^+^, while laminin‐α5^+^ capillaries were less depleted in anti‐Mi‐2^+^ DM patients’ biopsies (middle panel). Double immunofluorescence showed that atrophic muscle fibres co‐stained with neonatal myosin heavy chain (nMyHc) and VEGF (orange arrow) both in TIF‐1γ^+^ and in Mi‐2^+^ cases, however, not all regenerating myofibres were also VEGF^+^ (right panel)

To investigate protein localization of VEGF in the skeletal muscle specimens, we performed immunohistochemistry and immunofluorescence stainings. In anti‐TIF‐1γ^+^ DM patients’ muscles, VEGF expression was clearly enhanced in perifascicular areas, most evident on atrophic muscle fibers with a decreasing gradient towards the center of the fascicle (Figure 3). In contrast, anti‐Mi‐2^+^ DM patients showed‐in line with the RNA expression data‐less positive myofibers with a more diffuse distribution pattern (Figure 3). Double labeling of VEGF and laminin‐α5‐expressed around blood vessels‐identified severe loss of capillaries in the perifascicular area between the VEGF^+^ myofibers in anti‐TIF‐1γ^+^ patients’ skeletal muscles (Figure 3). These VEGF^+^ myofibers were identified as regenerating fibers by the co‐expression of the neonatal myosin heavy chain (nMyHc, Figure 3). Regular laminin‐α5‐positive capillaries were not severely diminished in anti‐Mi‐2^+^ DM patients’ muscles. Here, many perifascicular fibers showed sarcolemmal laminin‐α5 expression (Figure 3).

DDX58/RIG‐1 was found in the perifascicular area, weakly expressed on the sarcolemma of muscle fibers and on single immune cells in both subgroups without major differences (Figure S1). DDX58/RIG‐1 expression was not detected on CD68^+^ macrophages or CD8^+^ T‐cells (Figure S1).

### 3.4 RON/MSTR1 is mainly expressed on endothelial cells of intramuscular capillaries

Next, we investigated, which cells expressed RON/MST1R in our cohort. We identified CD31^+^/RON^+^ endothelial cells in intramuscular capillaries in both subgroups (Figure S2), whereas there was no co‐labeling of RON and PDGFRB^+^ pericytes (Figure S2). To further elucidate the expression of the phosphorylated form (Phospho‐RON, p‐RON), we performed various double immunofluorescence staining which revealed no co‐labeling of pRON and nMyHc^+^ regenerating fibers, MHC class II^+^ M1 or CD206^+^ M2 macrophages nor CD8^+^ T‐cells. We identified single CD4^+^/pRON^+^ cells in both subgroups (Figure S2).

## 4 DISCUSSION

Dermatomyositis‐specific autoantibodies such as anti‐TIF‐1γ have been shown to tightly correlate with organ involvement and malignancy in cancer‐associated myositis (2, 4, 5, 10). Thus, these autoantibodies are now recognized as useful biomarkers to stratify DM patients into clinical subgroups, and assessing the correct TIF‐1γ antibody status is of high importance to determine diagnostic procedures and prognosis.

The correlation of cancer and Mi‐2 autoantibodies, however, remains debated. While some groups reported no increased risk of cancer development in anti‐Mi‐2^+^ patients (38, 39, 40), other more recent studies on larger patients cohorts demonstrated a correlation of Mi‐2 antibody and CAM (41, 42). In our cohort, CAM was detected in 60% of anti‐TIF‐1γ‐ and 20% of anti‐Mi‐2‐associated DM patients, which (i) basically reflects results from other larger patient series (43, 44) and (ii) emphasized the need for personalized risk‐stratified cancer follow‐up for both DM subgroups.

We demonstrate that determining the NanoString nCounter PanCancer Immune Profiling Panel™ from skeletal muscle tissue may be alternatively used or in addition to distinguish both subgroups based on their distinct immune gene expression profiles. Moreover, our results suggest a specific expression profile of TIF‐1γ^+^/CAM+ cases. As a limitation of our study is the small sample size, this needs to be followed up in larger, prospective studies to further elucidate signaling pathways associated with cancer development and manifestation in these patients.

Both anti‐TIF‐1γ^+^ and anti‐Mi‐2^+^ patients’ skeletal muscle biopsies revealed strong dysregulation of immune response‐related genes compared to NDCs. In line with previous reports, these included well‐known type 1 IFN‐inducible genes such as *ISG15*, *ISG20*, *SIGLEC1*, *IRF7*, and *MX1* (45, 46, 47, 48, 49, 50). We also identified subgroup‐specific differences: TIF‐1γ^+^ patients showed upregulation of *HLA*‐*G*, *HLA*‐*DQA1*, and *BAGE*, the latter being a well‐known tumor antigen (51, 52, 53) present in many cancers (54). Dysregulation of HLA genes including *HLA*‐*DQA1*, *HLA*‐*A*, and *HLA*‐*G* are described in DM patients and HLA gene polymorphisms were shown to be susceptibility factors in myositis (55, 56).

In direct comparison to anti‐Mi‐2^+^ DM patients’ biopsies, anti‐TIF‐1γ^+^ patients’ biopsies showed an increased number of VEGF^+^ atrophic, nMyHc^+^ regenerating muscle fibers and a marked loss of perifascicular capillaries. The capillary drop out, a feature previously noticed in DM (45) may be caused by C5b‐9‐driven angiodestruction in TIF‐1γ^+^ patients (5, 57) and may well explain the significant upregulation of *VEGF* mRNA in the muscle tissue of TIF‐1γ^+^ patients in contrast to that in anti‐Mi‐2^+^ patients, which is most likely the result of increased levels of hypoxia in the perifascicular region (58, 59, 60, 61). Nevertheless, the function of VEGF is not limited to angiogenesis and vascular permeability (62), but also affects the function of immune cells and contributes to key aspects of tumor initiation and tumorigenesis (63). In fact, VEGF induces the expression of programmed cell death 1 ligand 1 (PD‐L1), an inhibitory ligand, which may lead to decrease the ability of the immune system to detect and eliminate tumor‐associated antigens (64). However, whether VEGF overexpression in the muscle tissue of TIF‐1γ^+^ patients contributes to tumor initiation or progression needs further investigation.

Retinoic acid‐inducible gene I (*DDX58/RIG‐1*) a cytosolic pattern recognition receptor, which is responsible for the type‐1 interferon (IFN1) response, was also found to be significantly upregulated in anti‐TIF‐1γ^+^ muscle biopsies in contrast to anti‐Mi‐2^+^. Immunohistochemically, the expression of RIG‐1 was found on the sarcolemma of muscle fibers in the perifascicular area and on single immune cells in both subgroups (anti‐Mi‐2^+^, anti‐TIF‐1γ^+^). *DDX58*/*RIG*‐*1* was shown to be directly involved in virus recognition and interferon production (47, 65, 66, 67). Nevertheless, it has been shown that increased RIG‐1 expression may be involved in limiting innate immune response and supporting tumor growth (67).

RON expression has recently been shown in a wide variety of human cancers (68) and is associated with malignant progression (69, 70), whereas in inflammation, it is described to suppress the inflammatory response (69). Analyzing the expression of MST1R/RON, showed a specifically upregulation in the muscle tissues of TIF‐1γ^+^ patients in contrast to those of NDCs.

We identified CD31^+^ endothelial cells as the major source of RON in both, anti‐TIF‐1γ^+^ and anti‐Mi‐2^+^ muscle biopsies. MST1R/RON induces molecular and cellular alterations (70), which may contribute to endothelial dysfunction and damage in DM, but this needs further investigation. Regulation of genes that are known to be associated with cancer (*VEGFA*, *DDX58*/*RIG1*, *MST1R*/*RON*, *BAGE*, and *TPTE*) in anti‐TIF‐1γ^+^ patients, and their expression on protein level, however, need further investigation as these genes are also involved in other biological processes apart from tumorigenesis.

Anti‐TIF‐1γ^+^ skeletal muscles showed compared to anti‐Mi‐2^+^, only scarce inflammatory infiltrates (1), which goes in line with the downregulation of different genes involved in immune response, such as *CD74* (HLA class II histocompatibility antigen gamma chain) (71), leucocyte differentiation antigen *CD84* (72, 73) as well as Chemokine (C‐C motif) ligand 5 (*CCL5*), which plays an active role recruiting leukocytes to inflammatory sites and which is also relevant to induce immune responses against tumors (74). Further downregulated genes were Integrin beta chain‐2 (*ITGB2*, CD18), which together with Integrin alpha L (*ITGAL*, CD11A, lymphocyte function‐associated antigen 1) form the lymphocyte function‐associated antigen‐1 (LFA‐1) playing a major role in neutrophil and T‐cell trafficking, extravasation, and emigration (75, 76).

Anti‐Mi‐2‐associated DM, in contrast, reflects a pathogenetically distinct subgroup, for which the specific type I interferon and inflammatory response was most prominent. From 109 upregulated genes in anti‐Mi‐2^+^ muscles compared to NDC muscles, 72 were identified to be IFN1‐inducible, which is histologically reflected by dense, mixed inflammatory infiltrates in the muscle biopsies. Therefore, the IFN signature in DM seems to be very prominent and pathogenically relevant especially in anti‐Mi‐2^+^ patients’ biopsies. Our results subsequently enlarge the current knowledge on DM subgroup‐specific deregulation of IFN1 pathways, which was previously investigated by Pinal‐Fernandez et al., but for only a very limited number of IFN1‐inducible genes (48).

Furthermore, our observation paves the way for JAK inhibitors as a treatment strategy to block type I interferon pathway activation in DM (47), which‐in light of our results‐may be more effective in anti‐Mi‐2^+^‐ than in anti‐TIF‐1γ^+^‐ associated DM.

We show that NanoString analysis is a very sensitive method (77, 78) to identify a distinct gene signature in skeletal muscle tissues of DM patients with anti‐TIF‐1γ or with anti‐Mi‐2 autoantibodies for diagnostic evaluation, cancer surveillance, and clinical follow‐up. In comparison, immunofluorescence or qPCR studies did not identify a distinct anti‐TIF‐1γ^+^‐specific biomarker that is associated with an increased risk of CAM showing that NanoString analysis is useful for better understanding of the DM etiology, prognosis, and identifying better treatment strategies.

## CONFLICT OF INTEREST

The authors report no disclosures relevant to the manuscript.

## AUTHOR CONTRIBUTIONS

Corinna Preusse, Werner Stenzel, and Josefine Radke designed the study concept. Pascale Eede, Kiara Freitag, Randi Koll, Waltraud Froehlich, Corinna Preusse, Udo Schneider, and Josefine Radke performed data analysis. Corinna Preusse and Josefine Radke wrote the manuscript. Anne Schänzer provided muscle biopsy samples and revised the manuscript. Lucie Heinzeling, Yves Allenbach, Olivier Benveniste, Hans‐Hilmar Goebel, and Werner Stenzel revised the manuscript and contributed to data analysis.

## Supporting information

Fig S1‐S2
**FIGURE S1** Histological and immunofluorescent staining of DDX58/RIG‐1 in DM patients’ skeletal muscle samples. DDX58/RIG‐1 was found in perifascicular areas on the sarcolemma of muscle fibres and single immune cells (left panel). Expression was not detected on CD68^+^ macrophages (middle panel) nor on CD8^+^ T‐cells (right panel)
**FIGURE S2** Immunofluorescent staining of RON/MST1R in DM patients’ skeletal muscle samples. (A) We identified single CD31^+^RON^+^ cells, while there was no co‐labelling between RON and PDGFRB^+^ pericytes or fibroblasts (left panel) or pRON and nMyHc^+^ regenerating fibres (middle planel) or MHC class II^+^ macrophages (right panel). (B) We revealed no co‐labelling of pRON and CD8^+^ T‐cells (left panel) or CD206^+^ M2 macrophages (middle panel). However, we identified single CD4^+^/pRON^+^ cells in both subgroups (right panel)

Table S1‐S2
TABLE S1 clinical information
TABLE S2 antibody information

Table S1‐S3
TABLE S3 NanoString results

## Data Availability

The authors of this manuscript state, that they have carefully documented data, methods, and materials used to conduct the research in the article. Data not provided in the article because of space limitations can be made available at the request of other investigators for purposes of replicating procedures and results. To our knowledge, there are no legal or ethical reasons or any embargoes on datasets, which may restrict this data availability policy.
