# Corrigendum

**DOI:** 10.1111/bpa.13053

**Published:** 2022-02-25

In this paper [1], the author name *Lucie Heinzeling* was incorrect and should have read *Lucie Heinzerling*.

The online version has been corrected.
