# Supplementary material for: NanoString technology distinguishes anti‐TIF‐1γ+ from anti‐Mi‐2+ dermatomyositis patients
Source: Brain Pathol. 2021 May 27;31(3):e12957. doi: 10.1111/bpa.12957 (PMC8412076; doi:10.1111/bpa.12957)
Supplement: Supplementary file 1 — Fig S1‐S2 FIGURE S1 Histological and immunofluorescent staining of DDX58/RIG‐1 in DM patients’ skeletal muscle samples. DDX58/RIG‐1 was found in perifascicular areas on the sarcolemma of muscle fibres and single immune cells (left panel). Expression was not detected on CD68+ macrophages (middle panel) nor on CD8+ T‐cells (right panel) FIGURE S2 Immunofluorescent staining of RON/MST1R in DM patients’ skeletal muscle samples. (A) We identified single CD31+RON+ cells, while there was no co‐labelling between RON and PDGFRB+ pericytes or fibroblasts (left panel) or pRON and nMyHc+ regenerating fibres (middle planel) or MHC class II+ macrophages (right panel). (B) We revealed no co‐labelling of pRON and CD8+ T‐cells (left panel) or CD206+ M2 macrophages (middle panel). However, we identified single CD4+/pRON+ cells in both subgroups (right panel) [file BPA-31-e12957-s002.docx]

NanoString technology distinguishes anti-TIF-1γ^+^

from anti-Mi-2^+^ dermatomyositis patients

Corinna Preuße^1,2,*^,Pascale Eede^1,*^, Lucie Heinzeling^3,4^, Kiara Freitag^1,5^, Randi Koll^1,6^, Waltraud Froehlich^3^, Udo Schneider^7^, Yves Allenbach^8^, Olivier Benveniste^8^, Anne Schänzer^9^, Hans-Hilmar Goebel^1^, Werner Stenzel^1^, and Josefine Radke^1,6,10,#^

^1^Department of Neuropathology, Charité-Universitätsmedizin Berlin, corporate member of Freie Universität Berlin, Humboldt-Universität zu Berlin, and Berlin Institute of Health (BIH), Berlin, Germany

^2^Department of Neurology with Institute for Translational Neurology, Münster University Hospital (UKM), Münster, Germany

^3^Department of Dermatology, University Hospital of Erlangen, Erlangen, Germany

^4^Department of Dermatology, LMU, Munich, Germany

^5^German Center for Neurodegenerative Diseases (DZNE) within the Helmholtz Association, Berlin, Germany

^6^German Cancer Consortium (DKTK), Berlin, Germany

^7^Department of Rheumatology and Clinical Immunology, Charité-Universitätsmedizin Berlin, corporate member of Freie Universität Berlin, Humboldt-Universität zu Berlin, and Berlin Institute of Health (BIH), Berlin, Germany

^8^Sorbonne Université, Assistance Publique – Hôpitaux de Paris, Inserm U974, Department of Internal Medicine and Clinical Immunology, Pitié-Salpêtrière University Hospital, Paris, France

^9^Department of Neuropathology, Justus Liebig Universität Giessen, Giessen, Germany

^10^Berlin Institute of Health (BIH), Berlin, Germany

* shared first authorship

# corresponding author

Dr. Josefine Radke

Department of Neuropathology

Charité-Universitätsmedizin Berlin

Virchowweg 15 / Charitéplatz 1


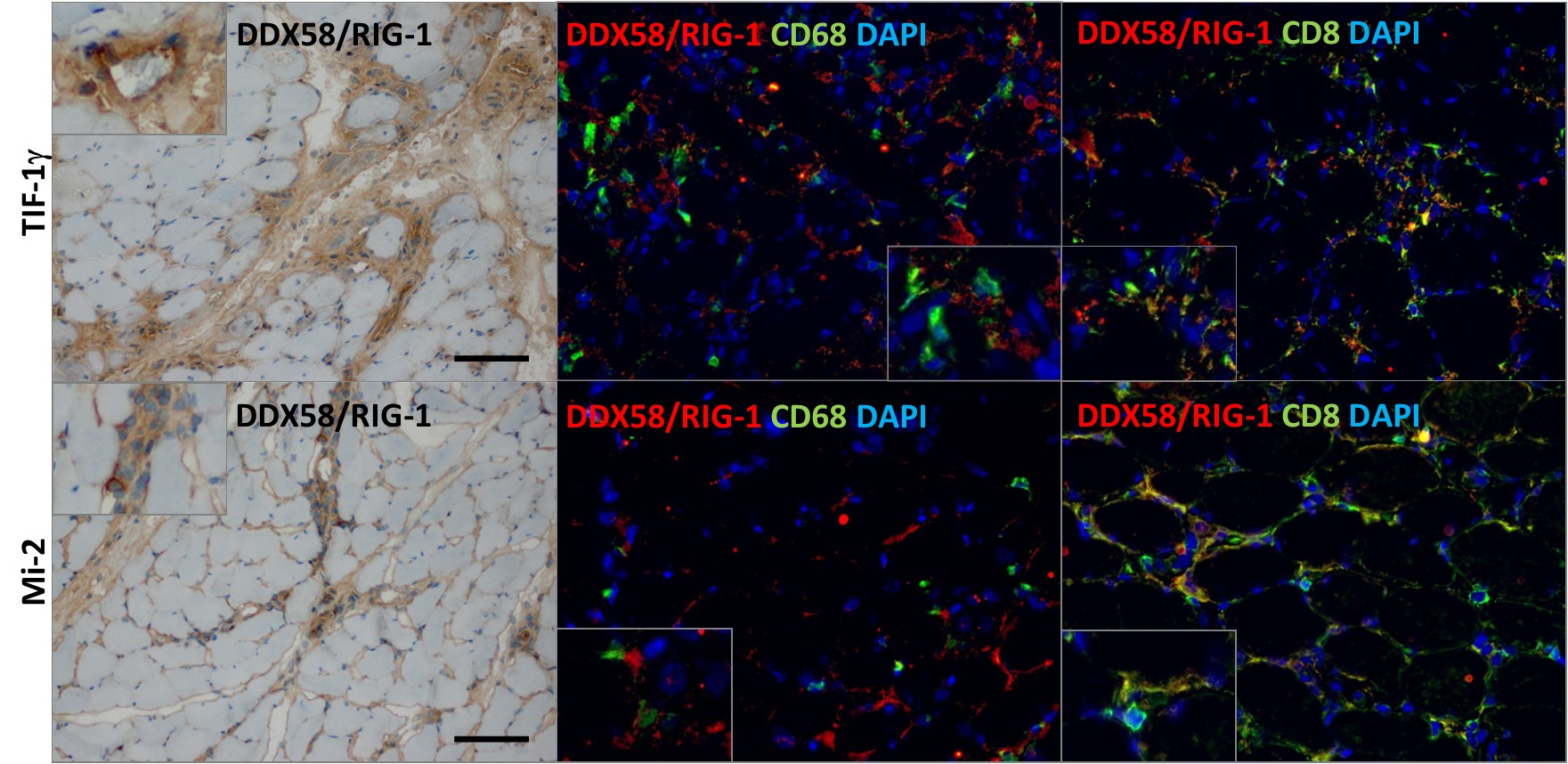


**Supplemental Figure 1:** Histological and immunofluorescent staining of DDX58/RIG-1 in DM patients’ skeletal muscle samples.

DDX58/RIG-1 was found in perifascicular areas on the sarcolemma of muscle fibres and single immune cells (left panel). Expression was not detected on CD68^+^ macrophages (middle panel) nor on CD8^+^ T-cells (right panel).


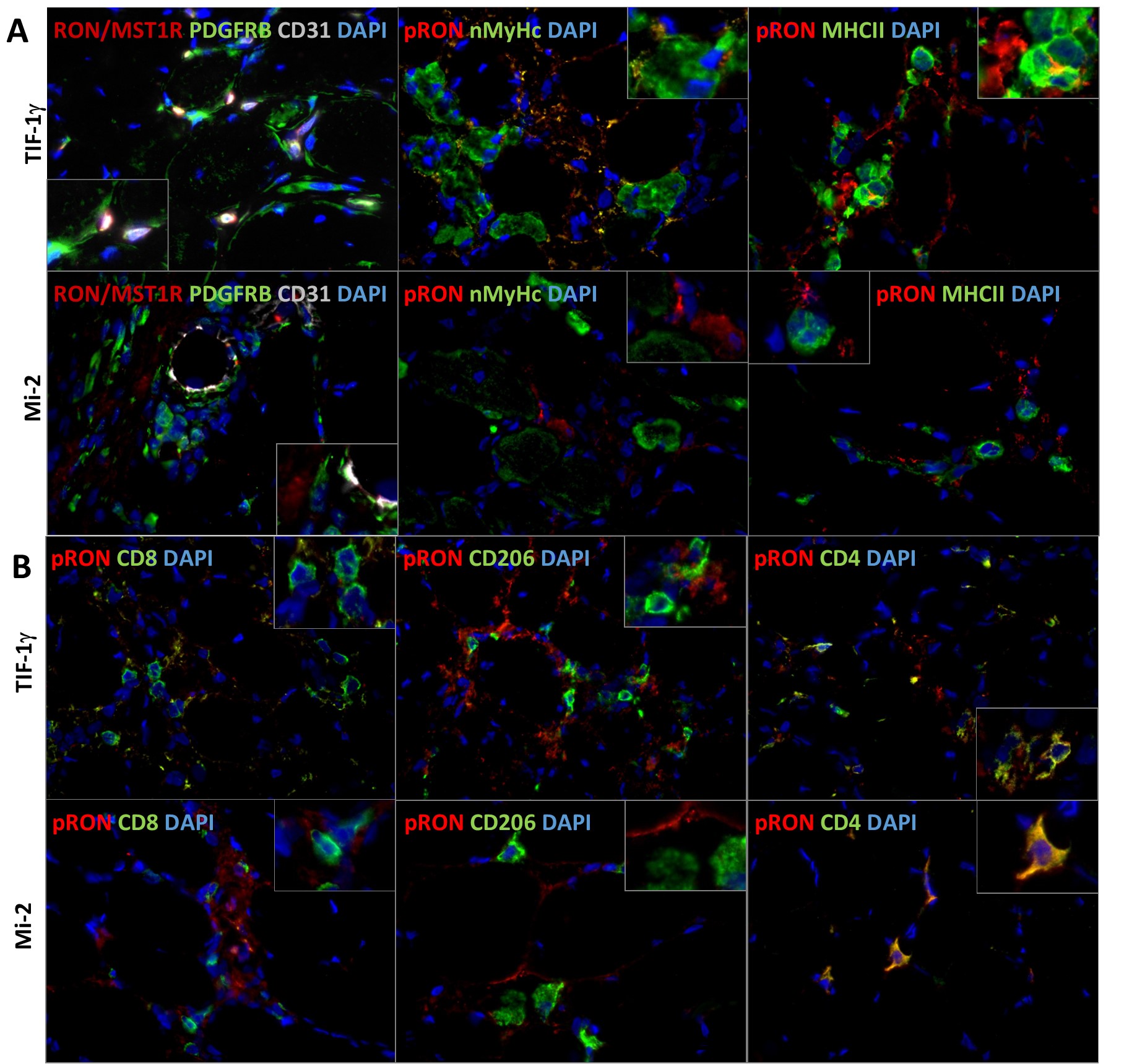


**Supplemental Figure 2:** Immunofluorescent staining of RON/MST1R in DM patients’ skeletal muscle samples.

**A**: We identified single CD31^+^RON^+^ cells, while there was no co-labelling between RON and PDGFRB^+^ pericytes or fibroblasts (left panel) or pRON and nMyHc^+^ regenerating fibres (middle planel) or MHC class II^+^ macrophages (right panel)

**B**: We revealed no co-labelling of pRON and CD8^+^ T-cells (left panel) or CD206^+^ M2 macrophages (middle panel). However, we identified single CD4^+^/pRON^+^ cells in both subgroups (right panel).

## 
