# Supplementary material for: NanoString technology distinguishes anti‐TIF‐1γ+ from anti‐Mi‐2+ dermatomyositis patients
Source: Brain Pathol. 2021 May 27;31(3):e12957. doi: 10.1111/bpa.12957 (PMC8412076; doi:10.1111/bpa.12957)
Supplement: Supplementary file 2 — Table S1‐S2 TABLE S1 clinical information TABLE S2 antibody information [file BPA-31-e12957-s003.docx]

Supplementary table 1: clinical information

|  |  | Mi-2 (n=15) | TIF-1γ (n=15) | NDC (n=8) |
| --- | --- | --- | --- | --- |
| age (mean ± SD) |  | 58.5 | 64.8 | 38.2 |
| sex | female | 73% (11) | 73% (11) |  |
|  | male | 27% (4) | 27% (4) |  |
| Biopsy location | M. deltoideus | 67% (10) | 47% (7) |  |
|  | other |  | 7% (1) |  |
|  | unknown | 33% (5) | 33% (5) |  |
| CK-values | normal | --- | 7% (1) | 100% (6) |
|  | >1-fold | 20% (3) | 27% (4) |  |
|  | >10-fold | 40% (6) | 13% (2) |  |
|  | unknown | 40% (6) | 53% (8) |  |
| symptoms | Muscle weakness | 67% (10) | 60% (9) |  |
|  | Muscle pain | 73% (11) | 27% (4) |  |
|  | Typical DM skin lesions | 67% (10) | 47% (7) |  |
|  | ILD | 7% (1) | --- |  |
|  | dysphagia | 13% (2) | 40% (6) |  |
|  | Unspecific complaints |  |  | 100% (6) |
| neoplasia | CAM+ | 20% (3)^¥^ | 60% (9)^§^ |  |
|  | CAM- | 7% (1)* | 0% (0) |  |
|  | No cancer^ǂ^ | 47% (7) | 27% (4) |  |
|  | unknown | 27% (4) | 13% (2) |  |
| Type of cancer |  |  |  |  |
| Immunosuppressant therapy after BX | Yes | 47% (7) | 40% (6) |  |
|  | No | 20% (3) | 13% (2) |  |
|  | unknown | 33% (5) | 47% (7) |  |
| clinical outcome | remission |  | 27% (4) |  |
|  | no change |  | 40% (6) |  |
|  | unknown | 100% (15) | 33% (5) |  |
|  | deceased | 7% (1) | 0% (0) |  |

CK = creatine kinase

CAM+ = cancer-associated myositis, defined as cancer occurring during the interval of 2y before until 3y after diagnosis of myositis

CAM- = patients that suffered from cancer more than 2y before or 3y after diagnosis of myositis (not myositis associated)

^ǂ^no cancer = patients that never sufferer from any cancer

*cancer type not yet described in the context of DM and therefore defined as CAM-, e.g. gastrointestinal stromal tumor (GIST)

^¥^type of malignancy: gynaecological cancer (ovary, uterus or breast; n=2), prostate carcinoma (n=1)

^§^type of malignancy: nasopharyngeal carcinoma (n=1), bladder cancer (n=2), gynaecological cancer (ovary, uterus or breast; n=5), colon carcinoma (n=1)

Supplementary table 2: antibody information

|  |  | Clone | Dilution | Company |
| --- | --- | --- | --- | --- |
| VEGF | Mouse anti human | C-1 | 1:100 | Santa Cruz Biotechnology |
| DDX58/RIG1 | Mouse anti human | 2M6F10 | 1:20 | Invitrogen |
| MST1R/RON | Mouse anti human | 1B5 | 1:50 | CellSignaling |
| pRON | Rabbit anti human | EPR5598 | 1:500 | Abcam |
| nMyHc | Mouse anti human | NB-MHCn | 1:20 | Novocastra |
| MHCII | Mouse anti human | CR3/43 | 1:100 | DAKO |
| Laminin a5 | Mouse anti human | 4C7 | 1:30.000 | Millipore |
| CD31 | Mouse anti human | 7C70A | 1:25 | DAKO |
| PDGFRß | Rabbit anti human | P20 | 1:30 | Santa Cruz Biotechnology |
| CD4 | Rabbit anti human | SP35 | Ready to use | Zytomed |
| CD8 | Mouse anti human | 144B | 1:100 | DAKO |
| CD68 | Mouse anti human | EBM1 | 1:100 | DAKO |
| CD206 | Mouse anti human | 7-450 | 1:500 | Acris |
